# The histologic diagnosis of usual interstitial pneumonia of idiopathic pulmonary fibrosis. Where we are and where we need to go

**DOI:** 10.1038/s41379-021-00889-5

**Published:** 2021-08-31

**Authors:** Maxwell L. Smith

**Affiliations:** Department of Laboratory Medicine and Pathology, Mayo Clinic Arizona, Scottsdale, AZ USA

**Keywords:** Respiratory tract diseases, Microscopy

## Abstract

In the 50 years since its inception by Dr. Liebow, the diagnosis of usual interstitial pneumonia (UIP) by pathologists has changed significantly. This manuscript reviews the progressive history of the histologic diagnosis of UIP and summarizes the current state of histologic UIP and its relationship to the clinical syndrome idiopathic pulmonary fibrosis (IPF). Fibrotic lung disease mimics of UIP/IPF are reviewed and pearls for distinguishing these diseases from UIP/IPF are provided. Strategies for increasing the value of histologic assessment of biopsies in the setting of pulmonary fibrosis are also discussed.

## Introduction

The pathologic diagnostic term usual interstitial pneumonia (UIP) has changed dramatically since its introduction in the late 1960s to the present day. It has evolved from a wide spectrum of acute on chronic lung disease without a well-defined correlated clinical syndrome to a highly specific histologic diagnosis strongly associated with the clinical syndrome idiopathic pulmonary fibrosis (IPF). This evolution of the diagnostic term UIP has created challenges in the pathologic community and in the application of clinical trial results to clinical practice. This review discusses the evolution of histologic UIP over the years and defines the current state of histologic UIP in 2021. Cases that may have similarities to histologic UIP, but not meeting current criteria for UIP, including fibrotic chronic hypersensitivity pneumonitis (fHP), connective tissue disease-associated chronic fibrosing interstitial lung disease (CTD-ILD), and advanced fibrotic nonspecific interstitial pneumonia (fNSIP), are highlighted. Challenges and implications with the most recent clinical guidelines for the diagnosis of histologic UIP, and the decreasing number of biopsies with histologic UIP are described. Finally, opportunities for adding further value to the pathologic interpretation of fibrotic interstitial lung disease are emphasized.

## Evolution of histologic usual interstitial pneumonia

UIP is a pathologic diagnostic term introduced in 1969 as part of the initial classification of interstitial pneumonias^1^. In this initial classification, if a biopsy did not show features of desquamative interstitial pneumonia, bronchiolitis obliterans interstitial pneumonia, lymphoid interstitial pneumonia, or giant cell interstitial pneumonia, it fell into the category of UIP. As described in the initial publication, UIP was a “highly variegated lesion with evidence of hyaline membrane formation and varying degrees of exudation…including protein and a great variety of cells and there is interstitial organization^1^.” In 1969, the natural course of UIP was described as beginning with epithelial necrosis, progressing through diffuse alveolar damage, and then either resolving or progressing to interstitial proliferation and eventually honeycomb “end-stage” lung. Notably absent from this initial description was a specific associated clinical syndrome with histologic UIP, the importance of fibroblast foci (FF) in establishing a diagnosis of UIP, and the importance of identifying histologic features that point to a specific etiology for the pulmonary fibrosis.

The importance of FF in the diagnosis of UIP was not recognized until 16 years later with the first publication linking UIP with FF activity^2^. It was not until the turn of the millennium when UIP was clarified to be a chronic fibrosing interstitial lung disease that was associated with the clinical syndrome IPF^3^. IPF was defined as the clinical diagnostic term only to be used in the setting of patients with chronic fibrosing lung disease and a surgical biopsy showing UIP. Contrary to the initial description of UIP, cases with histologic features of acute lung injury, including hyaline membranes and organizing pneumonia, were excluded from UIP, and designated acute interstitial pneumonia^3^. The vast array of clinical terms used for the idiopathic progressive fibrotic lung disease that showed histologic features of UIP underscores the challenges with nomenclature around the turn of the millennium^4^.

In 2011, the clinical practice guidelines for the diagnosis of IPF from the American Thoracic Society/European Respiratory Society/Japanese Respiratory Society/Latin American Thoracic Association (ATS/ERS/JRS/ALAT) fundamentally changed the work-up of patients with suspected IPF and also the structure of the histologic criteria for the pathologic diagnosis of UIP^5^. The 2011 guidelines were the first to introduce the concept of a multidisciplinary diagnosis of IPF in patients *without* a surgical lung biopsy (SLB) if the patient had the characteristic clinical presentation, and a high-resolution computed tomography (HRCT) scan showing radiologic UIP according to the guidelines. This has impacted the pathology communities experience with surgical lung biopsies in the setting of fibrotic lung disease (see section on “Surgical lung biopsies” below). In addition, the guidelines recognize the importance of linking the histologic diagnosis of UIP with the clinical syndrome IPF and have thus provided the ability to assign a probability score for UIP based on histologic features (UIP, probable UIP, possible UIP, and not UIP in 2011, revised to UIP, probable UIP, indeterminate for UIP, and alternative diagnosis in 2018)^6^.

The histologic guidelines were revised and updated in 2018^6^ (Table 1). In-depth analysis of these multidisciplinary guidelines reveals critical themes that are important for pathologists to understand. First, in assessing the structure of the criteria for histologic UIP, it becomes clear the goal is to only assign a diagnostic category of UIP in cases highly likely to represent IPF. Put another way, the clinical organizations are encouraging the pathology community to increase the specificity of histologic UIP for the clinical syndrome IPF. This statement is supported by the provisions of a probability score for UIP and the relegation of UIP secondary to another cause to the indeterminant category. Second, there is an emphasis on a detailed histologic examination of lung biopsies to evaluate for features that may suggest an etiology for the fibrotic lung disease other than IPF. The most common diseases in the differential diagnosis include CTD-ILD and fHP. The guidelines encourage a detailed search for lymphoid hyperplasia, chronic pleuritis, organizing pneumonia, acute lung injury, and secondary follicles (features suggesting CTD-ILD), as well as airway centricity, extensive peribronchiolar metaplasia (PBM), and granulomas (features suggesting fHP).

**Table 1:** 2018 ATS/ERS/JRS/ALAT guidelines for the histopathologic diagnosis of idiopathic pulmonary fibrosis and 2020 ATS/JRS/ALAT guidelines for the histopathologic diagnosis of fibrotic hypersensitivity pneumonitis.

	**UIP**	**Probable UIP**	**Indeterminate for UIP**	**Alternative diagnosis**
2018 ATS/ERS/JRS/ALAT Guidelines for UIP	• Dense fibrosis with architectural distortion (i.e., destructive scarring and/or honeycombing) • Predominant subpleural and/or paraseptal distribution of fibrosis • Patchy involvement of lung parenchyma by fibrosis • Fibroblast foci • Absence of features to suggest an alternate diagnosis	• Some histologic features from column 1 are present but to an extent that precludes a definite diagnosis of UIP/IPF AND • Absence of features to suggest an alternative diagnosis OR • Honeycombing only	• Fibrosis with or without architectural distortion, with features favoring either a pattern other than UIP or features favoring UIP secondary to another cause • Some histologic features from column 1, but with other features suggesting an alternative diagnosis	• Features of other histologic patterns of IIPs (e.g., absence of FF or loose fibrosis) in all biopsies • Histologic findings indicative of other diseases (e.g., hypersensitivity pneumonitis, Langerhans cell histiocytosis, sarcoidosis, LAM)
	**fHP**	**Probable fHP**	**Indeterminant for fHP**	
2020 ATS/JRS/ALAT Guidelines for Fibrotic Hypersensitivity Pneumonitis	• Chronic fibrosing interstitial pneumonia (architectural distortion, FF, honeycomb OR fNSIP) **OR** • Airway-centered fibrosis **AND** • Poorly formed nonnecrotizing granulomas • Absence of features that might suggest an alternate diagnosis (see third column)	• Chronic fibrosing interstitial pneumonia (architectural distortion, FF, honeycomb OR fNSIP) **OR** • Airway-centered fibrosis **AND** • Absence of features that might suggest an alternate diagnosis (see third column)	• Chronic fibrosing interstitial pneumonia (architectural distortion, FF, honeycomb OR fNSIP) **AND** • Absence of features that might suggest an alternate diagnosis - Plasma cells more common than lymphocytes - Lymphoid hyperplasia - Sarcoidal-like granulomas - Aspirated particles	

*UIP* usual interstitial pneumonia, *IPF* idiopathic pulmonary fibrosis, *ATS* American Thoracic Society, *ERS* European Respiratory Society, *JRS* Japanese Respiratory Society, *ALAT* Latin American Thoracic Society, *FF* fibroblast foci, *LAM* lymphangioleiomyomatosis, *fNSIP* fibrotic nonspecific interstitial pneumonia.

Over the past 50 years, the pathologic diagnostic term UIP has evolved from the “usual” type of interstitial lung disease that included both fibrotic and acute forms to a rigidly defined pathologic term that is strongly associated with the clinical syndrome IPF. The days of calling all surgical lung biopsies with pulmonary fibrosis UIP are gone and there needs to be a focus on searching for histologic features that suggest an etiology to the pulmonary fibrosis. In 2021, it is essential to consider the etiology of fibrosis in addition to the basic pathologic pattern because in many settings the etiology will outweigh the pattern of fibrosis in making treatment decisions. Within the pathology community, and even in the pulmonary pathology community, there are pathologists who are practicing at various stages of this evolution resulting in a lack of specificity for what a pathologic diagnosis of UIP implies. This lack of specificity is an opportunity for improvement.

## Histologic UIP/IPF in 2021

**Fig. 1:**
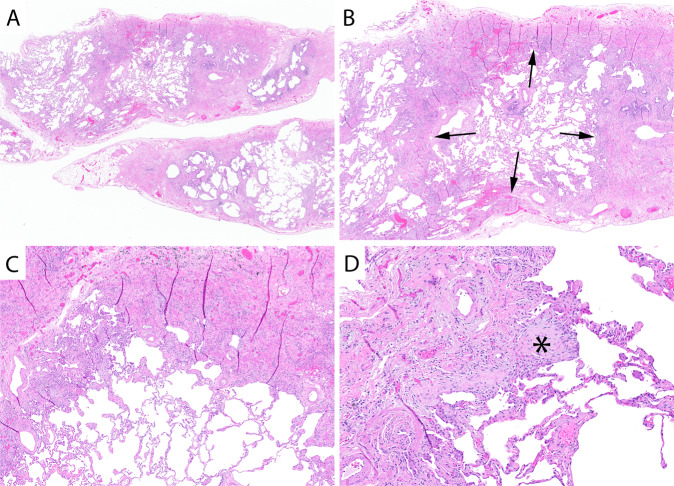
Usual interstitial pneumonia. Scanning magnification shows areas of advanced fibrosis with architectural distortion (**A**). Fibrosis at the periphery of the lobule (arrows) with sparring of the centrilobular regions (**B**). Sharp demarcation between the advanced fibrosis and the normal appearing alveolar walls (**C**). Evidence of active injury in the form of fibroblast foci (asterisks) (**D**).

As previously discussed, histologic UIP in 2021 has been refined to a more specific and criteria-driven diagnosis. Histologic UIP requires advanced fibrosis with architectural distortion (Fig. 1A). The distribution of fibrosis is particularly important in UIP. The fibrosis encountered in the clinical syndrome UIP/IPF begins at the periphery of the lobules and works its way toward the centrilobular regions. This results in peripheral “rings” or “donuts” of fibrosis in the subplural and paraseptal regions of the lobules (Fig. 1B). The fibrosis should be patchy with areas of advance fibrosis alternating with non-fibrotic lung parenchyma. Often the demarcation between the advanced fibrosis and non-fibrotic lung is sharply demarcated (Fig. 1C). Evidence of active injury in the form of FF is required. These FF are often at the interface between the advanced fibrosis and regions of uninvolved lung parenchyma (Fig. 1D). Finally, as mentioned previously, there should be an absence of histologic features to suggest an alternative etiology. These features include prominent airway-centered changes (bronchiolocentric distribution of fibrosis and/or extensive PBM), granulomas, areas of interstitial inflammation lacking associated fibrosis, prominent lymphoid hyperplasia including secondary germinal centers, marked chronic fibrous pleuritis, hyaline membranes, and organizing pneumonia^6^.

## Cases that are not histologic UIP in 2021

**Fig. 2:**
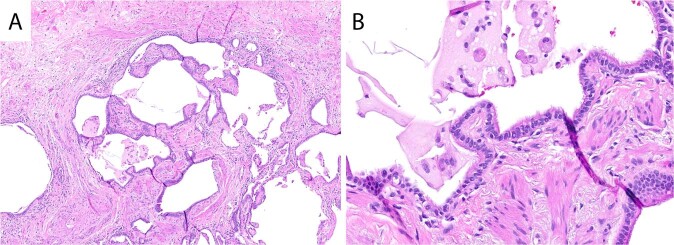
Honeycomb lung. Dilated cystic spaces embedded within advanced fibrosis (**A**). The cysts are lined by ciliated respiratory epithelium and the spaces are often filled with mucous debris (**B**).

Honeycomb lung is the most common scenario in which the category of probable UIP is encountered. Honeycomb lung is defined by the presence of cystically dilated spaces embedded within advanced fibrosis, lined by respiratory epithelium, and often filled with mucus debris (Fig. 2). Even in the setting of honeycomb lung, one should search the biopsy for histologic evidence of an alternative etiology. It is also important to remember that honeycomb lung represents end-stage pulmonary fibrosis and may even be secondary to a localized phenomenon, such as chronic infection.

There are three other common scenarios where a histologic diagnosis of UIP may be considered but the case is better classified as indeterminate for UIP or as an alternative diagnosis. These include fHP, CTD-ILD, and advanced fibrotic NSIP.

The ATS/JRS/ALAT recently published clinical guidelines for the diagnosis of fHP^7^. The criteria suggested for the histologic diagnosis of fHP are notably broad (Table 1). Histologic evidence of a chronic fibrosing interstitial pneumonia or airway-centered fibrosis is required. Also required is the presence of poorly formed nonnecrotizing granulomas and the absence of features to suggest an alternative diagnosis (plasma cells more common than lymphocytes, lymphoid hyperplasia, sarcoidal-like granulomas, and aspirated particles). Additional histologic features that may or may not be present include organizing pneumonia, chronic bronchiolitis, and a cellular interstitial infiltrate. The broad criterion underscores the histologic diversity encountered in fHP.

**Fig. 3:**
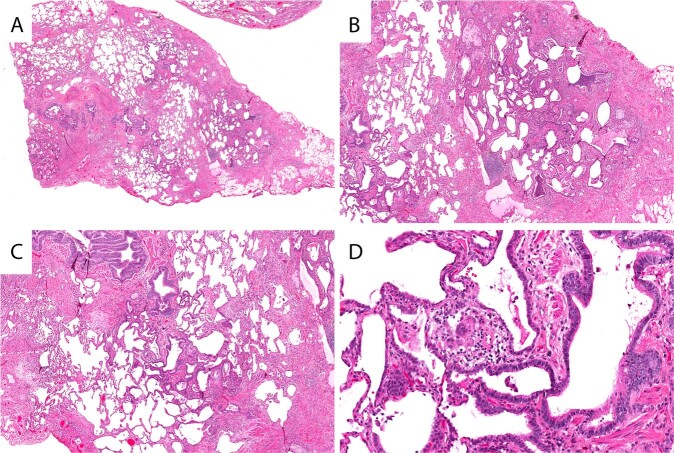
Fibrotic hypersensitivity pneumonitis. Patchy advanced pulmonary fibrosis (**A**) with areas of honeycomb (**B**) reminiscent of UIP/IPF. Peribronchiolar metaplasia that is out of proportion to the degree of scarring in the lobule (**C**). Poorly formed interstitial granuloma confirming the diagnosis of hypersensitivity pneumonitis (**D**).

When assessing biopsies of fHP, it is not uncommon to initially consider UIP because there is often fibrosis with a sense of heterogeneity from scanning magnification (Fig. 3A) and FF may be encountered (Fig. 3B)^8^. However, cases of fHP have more frequent bronchioles with PBM, more extensive PBM (Fig. 3C), and poorly formed granulomas (Fig. 3D)^9^. Biopsies with features seen in Fig. 3 would meet criteria for fHP and should be classified as Alternative Diagnosis using the 2018 UIP criteria.

**Fig. 4:**
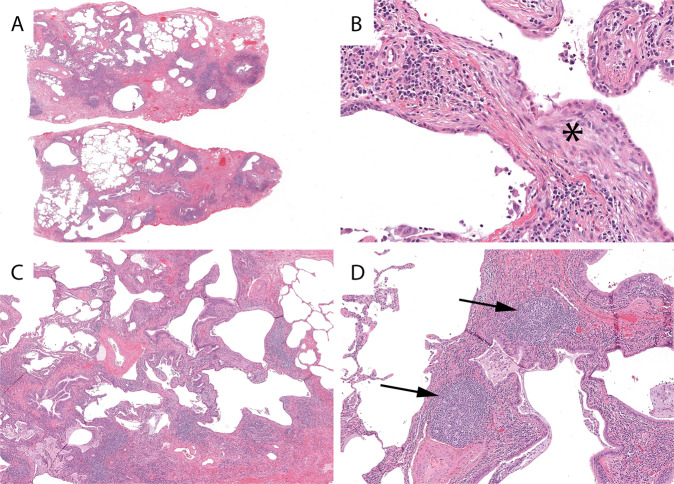
Connective tissue disease-associated fibrosing interstitial lung disease. Patchy advanced pulmonary fibrosis with areas of possible sparing from low power (**A**) and fibroblast foci (asterisks) (**B**) reminiscent of UIP/IPF. Lymphoplasmacytic infiltrates in the “normal” alveolar walls (**C**). Numerous lymphoid follicles including some with germinal centers (arrows) (**D**).

CTD-ILD can result in a spectrum of patterns of pulmonary fibrosis^10,11^. On their surface, some cases can be quite similar to UIP/IPF with regard to the distribution of the fibrosis and presence of FF (Fig. 4A, B). However, they are distinguished from UIP/IPF by the presence of dense lymphoplasmacytic infiltrates, lymphoid hyperplasia, presence of secondary lymphoid follicles, and chronic fibrosing pleuritis. Particular attention should be given to the areas of seemingly normal appearing lung from scanning magnification. In CTD-ILD they will often show a subtle but definitive lymphoplasmacytic infiltrate (Fig. 3C). Honeycomb fibrosis of any type can attract lymphoid aggregates, but numerous aggregates and ones with secondary follicles should raise suspicion for CTD-ILD (Fig. 4D). Polyps of organizing pneumonia are frequently encountered in cases of CTD-ILD. Cases with the changes encountered in Figure D should be assigned an Alternative Diagnosis using the 2018 UIP guideline criteria.

**Fig. 5:**
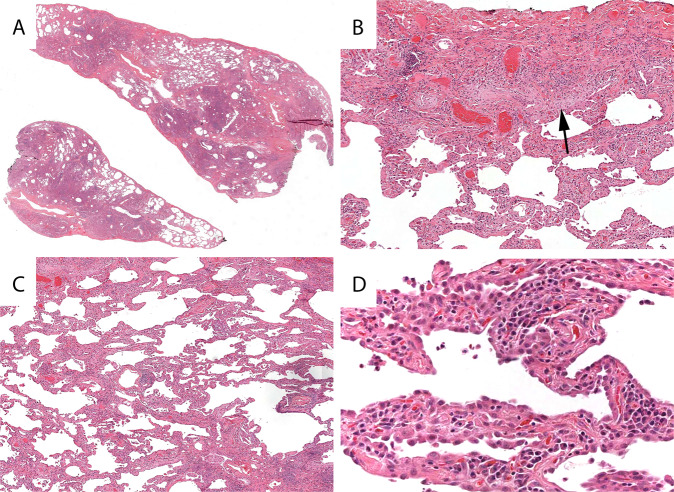
Fibrotic nonspecific interstitial pneumonia. Pulmonary fibrosis with apparent geographic heterogeneity from low power (**A**) and fibroblast foci (arrow) (**B**) reminiscent of UIP/IPF. Subtle nonspecific interstitial pneumonia infiltrates in the less fibrotic areas (**C**). Lymphoplasmacytic interstitial infiltrates (**D**).

Classic NSIP is described as having variable amounts of interstitial inflammation and fibrosis with a uniform appearance with organizing pneumonia and honeycomb fibrosis being inconspicuous or absent. This uniform appearance is in reference to the *temporal* aspects of the histology, not the uniform involvement of all sampled lung tissue. Normal areas of lung may be present in biopsies of NSIP^12^. Fibrotic NSIP is characterized by interstitial thickening by uniform fibrosis of the same age usually preserving the alveolar architecture with varying amounts of cellular inflammation^13^. Some cases of *advanced* fNSIP may be confused with UIP/IPF due to the presence of geographic variability and “enlarged air-spaces” easily interpreted as honeycombing^13^. Despite the presence of thousands of figures showing the uniform fibrosis of fNSIP in textbooks and manuscripts the reality is that most cases have some variability from section to section and lobe to lobe. It is rare to encounter clinical cases with the perfect uniformity seen in textbooks (Fig. 5A). Enlarged air-spaces mimicking honeycomb change, and even true honeycomb change, may be encountered in up to 22% of fNSIP cases^14,15^. The distinction of fNSIP from UIP/IPF is further complicated by the presence of FF in up to 20% of cases (Fig. 5B)^14^. Therefore, the presence of honeycomb change and FF *alone* should *not* be used as diagnostic criteria for UIP/IPF. A careful search of these biopsies in the “normal” or less involved areas often reveals lymphoplasmacytic infiltrates creating a subtle NSIP-like pattern (Fig. 5C, D). Biopsies such as this should be diagnosed as fNSIP and classified as an Alternative Diagnosis according to the 2018 UIP guideline criteria.

Although the salient histologic features distinguishing fHP, CTD-ILD, and fNSIP from UIP/IPF are provided above, it should be noted that in each case not all features may be present or additional confounding features may be present, thus creating histologic uncertainty. For example, some cases of fHP may not have characteristic interstitial granulomas^16^ or some cases of IPF may have increased interstitial inflammation, especially in areas of scarring^6^. This uncertainty is the likely driver for the guidelines providing pathologists with options to give the likelihood of a UIP diagnosis. Multidisciplinary diagnosis, incorporating clinical, radiologic, and pathologic features remains the gold standard of clinical ILD diagnosis and can serve to clarify the diagnosis in cases with histologic uncertainty^6,7^.

## Challenges associated with the 2018 guidelines

Challenges associated with the 2018 ATS/ERS/JRA/ALAT guidelines have been reviewed previously^17,18^. Briefly, there are three major challenges associated with the implementation of these guidelines in pathology practice. First, there are multiple different UIP based guidelines^6,19^ as well as different guidelines for other fibrotic lung diseases^7^, leading to confusion as to when and how to use which guideline. Is it time for a guideline on how to utilize the various guidelines? Second, the guidelines imply a siloed approach to radiologic and histologic designation of the guideline category and no suggestion is made as to how a pathologist should handle clinical and radiologic information available at the time of the interpretation. Finally, there is no guidance on how to weigh each individual histologic feature versus the using an amalgam of the collective features seen on a biopsy.

The guideline reference to “UIP secondary to another cause” deserves a specific comment. The concept of histologic UIP resulting from an etiology other than IPF generates a significant amount of confusion for our clinical colleagues as most clinicians will interpret a UIP diagnosis from pathology as synonymous with IPF. Some authors prefer to avoid the phrases “UIP secondary to another cause” and “secondary UIP” if the histologic features are suggestive of an etiology other than IPF.

## Reasons for the decrease in surgical lung biopsies in fibrotic lung disease

SLB is considered the gold standard in the histologic diagnosis of ILD. However, both the frequency of SLB and the frequency of UIP encountered on SLB have decreased over time^20^. There are several factors that have contributed to this decline in frequency. First, SLB carries significant morbidity and mortality^21^. In the largest analysis to date, the rate of mortality for elective and non-elective SLB was 1.7% and 16%, respectively^22^.

Second, a molecular classifier based on transbronchial biopsy material has been developed that correlates an RNA molecular signature with a SLB histopathologic diagnosis of UIP^23^. The Envisia Genomic Classifier produces a binary result, either UIP or not UIP. Subsequent studies have confirmed the accuracy and reproducibility of the classifier for the identification of biopsy-proven UIP with a sensitivity of 60.3% and a specificity of 92.1%^24^. However, caution should be exercised regarding the molecular classifier as causes of a UIP pattern aside from IPF were not excluded. Essentially, a positive molecular classifier result is compatible with advanced pulmonary fibrosis, a fact likely known based on the HRCT findings already. The test was not developed to specifically identify IPF versus other causes of pulmonary fibrosis.

Third, cryobiopsy has been introduced as an alternative technique for obtaining lung tissue for the diagnosis of interstitial lung disease^25^. Prior to the introduction of cryobiopsy, the only options for obtaining tissue for ILD diagnosis were transbronchial forceps biopsy (TBBX) and SLB. It is widely recognized that the vast majority of ILD cannot be accurately and reproducibly diagnosed on TBBX^26^. Diseases with centrilobular distribution, such as sarcoidosis, berylliosis, lymphangitic carcinomatosis, and hypersensitivity pneumonitis, show the highest yield for diagnosis on TBBX. TBBX cannot be used for the diagnosis of UIP/IPF^5,27^. These limitations of TBBX, combined with the risk profile of SLB motivated the development of the cryobiopsy technique. As compared to TBBX, cryobiopsy generates larger tissue fragments (>1 cm) and the procedure results in less crush artifact. In theory, both features could improve the diagnostic yield on cryobiopsy. However, the studies on the effectiveness of cryobiopsy have shown mixed results regarding diagnostic accuracy^28,29^. Nevertheless, the CHEST practice guidelines suggest cryobiopsy as a reasonable alternative to SLB^25^.

Fourth, the introduction of the practice guidelines for IPF in 2011, and continued in 2018, allow for the diagnosis of IPF in the correct clinical and radiologic setting, without a SLB^5,6^. This has changed the pretest probability for UIP on surgical lung biopsies in the setting of fibrotic lung disease. Only the cases with unusual clinical presentations and HRCT findings that are not classic for UIP/IPF end up meeting criteria for SLB.

Finally, over the past few years, there has been a shift to the progressive pulmonary fibrosis phenotype concept^30^. Within this concept, perhaps the specific etiology of the fibrosis is less important to determine, and it is more important to identify the patients who will progress clinically. Anti-fibrotic medications could then only be used in these patients. This concept introduces several challenges to the field of fibrotic interstitial lung disease and may be a symptom of pathologists’ lack of specificity regarding the use of the term UIP over the years. It is also based on access to anti-fibrotic medications that are costly, have a high side effect profile, and only decrease the rate of decline of forced vital capacity^31–33^. Further pivoting in the direction of the progressive pulmonary fibrosis concept has the potential to risk delaying the development of etiologic-based treatment modalities in the field for years.

## Potential avenues to increase the value of surgical lung biopsy interpretation

The field of pulmonary pathology has a number of avenues it can address synchronously to increase the clinical value of the SLB in the setting of fibrotic lung disease. First, the field is in need of standardizing the histologic assessment. Because of the significant changes in the histologic diagnosis if UIP/IPF over the past 20 years, there are a variety of practice habits currently in place. Some pathologists have a low threshold for diagnosing UIP on biopsy while others feel the current guidelines are so restrictive that a UIP diagnosis is never made. Second, as mentioned above, there are several issues associated with our current available guideline criteria that need to be addressed. Addressing some of the issues has the potential to help standardize the histologic assessment as well. Third, contrary to several other non-neoplastic fibrotic diseases, there is no histologic grading or staging for IPF. Aside from making the pattern-based diagnosis of UIP, pathologists do no supply clinical elements of disease activity (grade) or the degree of fibrosis (stage). It may be that the HRCT is best positioned to stage the entire lung parenchyma^34^, but perhaps there are histologic elements in the biopsies from patients with advanced pulmonary fibrosis that could correlate with treatment or prognosis aside from simply UIP. Finally, and most exciting, the field of pathology is on the verge of a digital revolution, enhanced by evolving artificial intelligence algorithms (AI)^35,36^. In addition to our pathologist interpretation, we will have the ability to extract a number of quantitative data elements that may have diagnostic, prognostic, or therapeutic significance. A recent study used AI to identify FF and inflammatory cells in biopsies from a well-characterized cohort of IPF patients. They found increased FF activity and decreased inflammation to be associated with a worse prognosis^37^. While FF and inflammation having a prognostic value is not a new concept, the ability to interrogate these histologic findings in a reproducible way without interobserver variability has the potential to add significant value to histologic analysis in ILD^38–40^. There is a goldmine of quantitative buried in the SLB and we are just now beginning to systematically mine for it.

## Conclusion

Over the past 50 years, the concept of UIP has shifted dramatically. UIP has evolved from a mixture of acute and chronic interstitial lung disease without a clinical correlate in the late 1960s to a highly specified histopathologic diagnosis in the present state, strongly associated with the clinical syndrome IPF. This evolution has stressed pathologists ability to provide useful and reproducible pathologic information beyond the diagnostic term UIP. This reviewed outlined the present-day diagnostic criteria for UIP and shared several cases that may be mistaken for UIP that actually represent an alternate diagnosis. There are many opportunities for the pulmonary pathology field to continue to contribute to the advancement of the diagnosis and treatment of patients with interstitial lung disease.

## Data Availability

Data sharing is not applicable to this article as no datasets were generated or analyzed during the current study.
